# Towards the preferred stimulus parameters for distortion product otoacoustic emissions in adults: A preliminary study

**DOI:** 10.4102/sajcd.v65i1.585

**Published:** 2018-07-16

**Authors:** Lucretia Petersen, Wayne J. Wilson, Harsha Kathard

**Affiliations:** 1 Division of Communication Sciences and Disorders, Department of Health and Rehabilitation Sciences, Faculty of Health Sciences, University of Cape Town, South Africa; 2 School of Health and Rehabilitation Sciences, Faculty of Health and Behavioural Sciences, University of Queensland, Australia

## Abstract

**Background:**

Although distortion product otoacoustic emissions (DPOAEs) are useful in evaluating cochlear outer hair cell function, determining the optimal stimulus parameters could result in a more reliable, sensitive and specific diagnostic tool across the range of DPOAE applications.

**Objectives:**

To identify which stimulus parameters warrant further investigation for eliciting the largest and most reliable DPOAEs in adult humans.

**Method:**

A single group, repeated measures design involving a convenience sample of 20 normal-hearing participants between 19 and 24 years of age.

**Results:**

Descriptive statistics and mixed model analyses suggested L_1_/L_2_ intensity levels of 65/65 dB sound pressure level (SPL) and 65/55 dB SPL, and *f*_*2*_*/f*_*1*_ ratios of 1.18, 1.20 and 1.22 elicited larger and more reliable DPOAEs in both ears.

**Conclusion:**

Further investigation of the 65/65 dB SPL and 65/55 dB SPL intensity levels and the 1.18, 1.20 and 1.22 *f*_*2*_*/f*_*1*_ ratios is warranted to determine the stimulus parameters for eliciting the largest and most reliable DPOAEs in adult humans across the range of DPOAE applications.

## Introduction

Distortion product otoacoustic emissions (DPOAEs) are sounds emitted from the cochlea in response to two simultaneously presented tonal stimuli. These stimuli have levels designated as L_1_ and L_2_ and frequencies designated as *f*_*1*_ and *f*_*2*_. The sensitivity of DPOAEs to outer hair cell dysfunction in the cochlea has seen them successfully used in a variety of clinical and research applications, such as newborn hearing screening, diagnostic audiological assessment, ototoxicity monitoring and the study of cochlear mechanics (Dhar & Hall, 2012; Hood & Berlin, 2002).

The successful use of DPOAEs in a range of applications suggests that their optimal stimulus parameters have been determined. This is not the case (Petersen, Wilson, & Kathard, 2017). Instead, the DPOAE level has been found to depend on varying combinations of stimulus parameters, including *f*_*1*_ and *f*_*2*_ frequencies, *f*_*2*_*/f*_*1*_ ratio, L_1_ and L_2_ intensity levels and L_1_/L_2_ level separation (Prieve & Fitzgerald, 2015). Furthermore, DPOAEs have been elicited using a wide range of stimulus parameters, including *f*_*2*_*/f*_*1*_ ratios from 1.03 to 1.79 and L_1_/L_2_ combinations ranging from 30/30 dB sound pressure level (SPL) to 85/85 dB SPL (Petersen et al., 2017). Dreisbach and Siegel (2001) added to this complexity by reporting that the optimal *f*_*2*_*/f*_*1*_ ratio varies as a function of *f*_*2*_ frequency, with lower *f*_*2*_*/f*_*1*_ values eliciting higher DPOAE levels at higher *f*_*2*_ frequencies and vice versa.

Recommended stimulus parameters have also varied depending on the application. For diagnostic purposes and/or ototoxicity monitoring, *f*_*2*_*/f*_*1*_ ratios have ranged from 1.20 to 1.22 and L_1_/L_2_ combinations from 45/35 dB to 65/55 dB SPL (Dhar & Hall, 2012; Hall, 2000). Hall (2000) also reported that for cochlear lesions, decreasing the stimulus levels improved DPOAE sensitivity, whereas increasing the stimulus levels improved DPOAE specificity. For screening applications, an *f*_*2*_*/f*_*1*_ ratio of 1.20 has often been recommended with L_1_/L_2_ combinations of either 65/55 dB SPL or 65/65 dB SPL (Dhar & Hall, 2012; Hall, 2000). Other recommendations have included an L_1_/L_2_ combination of 65/55 dB SPL for its reported twofold advantage of producing a higher DPOAE level with an improved sensitivity to cochlear dysfunction, whereas the use of L_1_/L_2_ combinations above 70/70 dB SPL has been discouraged to avoid possible response artifact that can be mistaken for DPOAEs and confusion over the source of the resulting DPOAEs (Dhar & Hall, 2012).

The variation in stimulus parameters used to elicit DPOAEs highlights the continuing need to determine the optimal DPOAE stimulus parameters across all its applications. This search needs to be driven by sound research based on a continuum of evidence that considers two factors: the cyclical nature of knowledge creation and the quality of existing evidence informing clinical practice. The cyclical nature of knowledge creation, especially in the case of clinical practice, refers to the cycle of developing theories that are then tested by research to develop new knowledge. This new knowledge is then applied to clinical settings where it is used to refine existing theories or propose new ones (Schmidt & Brown, 2015). Patient care is improved through the repeating nature of this cycle and its ability to generate ever-changing scientific knowledge. In the case of DPOAEs, the quality of existing evidence informing clinical practice refers to the following questions: ‘how rigorous is the research investigating DPOAE stimulus parameters?’ and ‘how strong is the evidence for the stimulus parameters currently used in the clinical setting?’ Asking such questions seeks to strengthen clinical practice and expand current evidence bases (Puddy & Wilkins, 2011).

In response to the above call, the authors of this study recently began to search for the optimal stimulus parameters for eliciting DPOAEs from human adults for clinical applications, especially to assist with early identification of cochlear damage in individuals receiving ototoxic treatment for multi-drug-resistant tuberculosis. This search began with a systematic review (Petersen et al., 2017) that first asked: ‘what is an “optimal” DPOAE?’ Factors such as the clinical value of DPOAE level, signal-to-noise ratio (SNR), reliability, sensitivity and specificity to cochlear dysfunction were considered, as well as confounds such as the high number of DPOAE parameters open to manipulation, the small effect sizes of changing some parameters, the physiological processes represented by DPOAEs and the high intersubject variability seen in DPOAEs. The review then examined 47 DPOAE studies that had met the inclusion criteria for the systematic review. Of these, 33 studies met the inclusion criteria to examine the influence of intensity and/or frequency ratio on the DPOAE level. Most of the studies were found to have small sample sizes (often fewer than 10 participants) and/or to have manipulated only one set of stimulus parameters (18 having manipulated L_1_/L_2_ levels at a fixed *f*_*2*_*/f*_*1*_ ratio, or vice versa). Of the remaining 15 studies that manipulated both intensity and frequency ratio parameters, 8 studies used 15 participants or fewer. Ten of the 15 studies had only used descriptive statistics when reporting their results, leaving open the possibility that any observed differences had occurred by chance alone (interestingly, this limitation was seen in two seminal and highly cited papers on DPOAE stimulus parameters by Gaskill and Brown [1990] and Harris, Lonsbury-Martin, Stagner, Coats and Martin [1989]). Petersen et al. (2017) concluded that although some parameters are commonly used to elicit DPOAEs, only their effects on DPOAE level have been considered in limited detail (with their effect of DPOAE SNR, reliability and sensitivity and specificity to cochlear dysfunction being largely ignored), and the optimal parameters for eliciting DPOAEs in adult humans in clinical applications have yet to be determined (Petersen et al., 2017).

This study sought to expand on the findings of Petersen et al. (2017) towards a final determination of the optimal stimulus parameters for eliciting DPOAEs from human adults for clinical diagnostic applications. It considered a wide range of commonly used stimulus parameters from those reported by Petersen et al. (2017) but expanded their investigation by systematically manipulating both *f*_*2*_*/f*_*1*_ ratios and L_1_/L_2_ levels simultaneously. This study was limited to measuring the effect of stimulus parameters on DPOAE level and reliability (and not SNR or sensitivity and specificity to cochlear dysfunction) in adult humans with normal hearing, to manage the total number of variables under examination.

## Method

### Research design

A single group, repeated measures design was used for this study. This design was deemed appropriate to examine the influence of intensity and frequency ratio stimulus parameters on DPOAE levels.

### Participants

Twenty normal-hearing adult participants (15 female, 5 male, aged 19 to 24 years) were conveniently sampled from the staff and student population of the University of Cape Town, South Africa. These participants had no obvious outer ear or tympanic membrane abnormalities on otoscopy, had hearing thresholds ≤ 15 dB HL at octave frequencies from 0.25 to 8 kHz on pure tone audiometry (Clark, 1981), had middle ear pressure and compliance within normal limits -(Grason-Stadler, 2001) on tympanometry, had no self-reported history of ear events that could affect DPOAE recordings and had passed a DPOAE screening assessment.

### Protocol

Participants were initially screened for inclusion in the study using a live voice interview and a commercially available otoscope, audiometer, tympanometer and DPOAE device (GSI Audera 2.7, Version C). To pass the DPOAE screening, the participants had to show 2*f*_*1*_-*f*_*2*_ DPOAEs at least 3 dB above the noise floor at *f*_2_ frequencies 2, 4 and 8 kHz to tonal stimuli with an *f*_*2*_*/f*_*1*_ ratio of 1.2 and an L_1_/L_2_ setting of 65/55 dB SPL. All initial testing was conducted in a sound-treated booth meeting South African National Standards (2006).

Distortion product otoacoustic emissions testing was conducted in a quiet room with background noise levels < 55 dB A as measured using a Brüel & Kjær 2238 class 1 handheld sound level meter. The 2*f*_*1*_-*f*_*2*_ DPOAE measurements were obtained from each ear of each participant using the following stimulus parameters: *f*_*2*_*/f*_*1*_ ratios – 1.18, 1.20, 1.22, 1.24, 1.26 and 1.28; L_1_/L_2_ settings – 65/65 dB SPL, 65/55 dB SPL, 60/45 dB SPL, 60/53 dB SPL and 55/40 dB SPL; and *f*_*2*_ frequencies: 2003 Hz, 2519 Hz, 3178 Hz, 3996 Hz, 5000 Hz, 6996 Hz and 8003 Hz. To mitigate potential order effects, a single sequence of stimulus parameters was set, and each participant was started at a different point in this sequence. The order of ear testing was reversed for each sequential participant. The 2*f*_1_-*f*_2_ DPOAEs were sampled until at least one of the two stopping rules was met: (1) the noise floor at the distortion product frequency was less than -10 dB SPL or (2) until 32 s of artifact-free sampling had been averaged (Dille et al., 2010). Participants were seated in a comfortable chair and were instructed to remain still and quiet during the DPOAE test procedure with breaks provided as required. The DPOAE test time per participant was approximately 90 min per test occasion. Each participant underwent DPOAE testing on two occasions 24 h – 48 h apart.

### Data collection

The following DPOAE data were recorded from each participant for each set of stimulus parameters at each *f*_2_ frequency on each test occasion: absolute level of DPOAE, absolute level of the noise floor and the DPOAE SNR, calculated as the absolute level of the DPOAE minus the level of the noise floor.

### Data analysis

All DPOAE data were found to meet parametric assumptions following examination of the histograms of these data, box-and-whisker plots and Q–Q plots (data not shown). Descriptive statistics were calculated for all DPOAE measures, and correlation analyses were conducted to determine if the DPOAE results for the left and right ears were related. As these analyses showed significant correlations in DPOAE results between the ears, all further analyses of the DPOAE data were conducted for each ear separately.

Two sets of linear mixed model analysis were conducted at the 5% significance level on the DPOAE data for each *f*_*2*_ value separately. Each set of analyses considered DPOAE amplitudes as dependent variables, the stimulus level combinations and frequency ratios as fixed effect independent variables and the participants as a random effect independent variable. The first set of analyses sought to identify the presence of any main effects of level settings (L_1_/L_2_ in dB SPL) for all *f*_*2*_*/f*_*1*_ settings combined, and any main effects of frequency ratio settings (*f*_*2*_*/f*_*1*_) for all L_1_/L_2_ settings combined. The second set of analyses sought to identify the presence of any main effects of the combined level (L_1_/L_2_ in dB SPL) and frequency ratio (*f*_*2*_*/f*_*1*_) settings.

Finally, two-way mixed model intraclass correlation coefficient (ICC) analyses for absolute agreement were conducted at the 5% significance level on the DPOAE data for each *f*_*2*_ value separately to determine the level of agreement (reliability) of the absolute levels of the DPOAE recordings from the first to the second assessment occasions for each combined level (L_1_/L_2_ in dB SPL) and frequency ratio (*f*_*2*_*/f*_*1*_) setting separately.

All statistical analyses were conducted using IBM SPSS Statistics versions 23 and 24 (64-bit edition).

## Ethical consideration

Unconditional ethical clearance was granted to conduct the study by the Faculty of Health Sciences Human Research Ethics Committee (HREC/REF: 512/2013).

## Results

**FIGURE 1:**
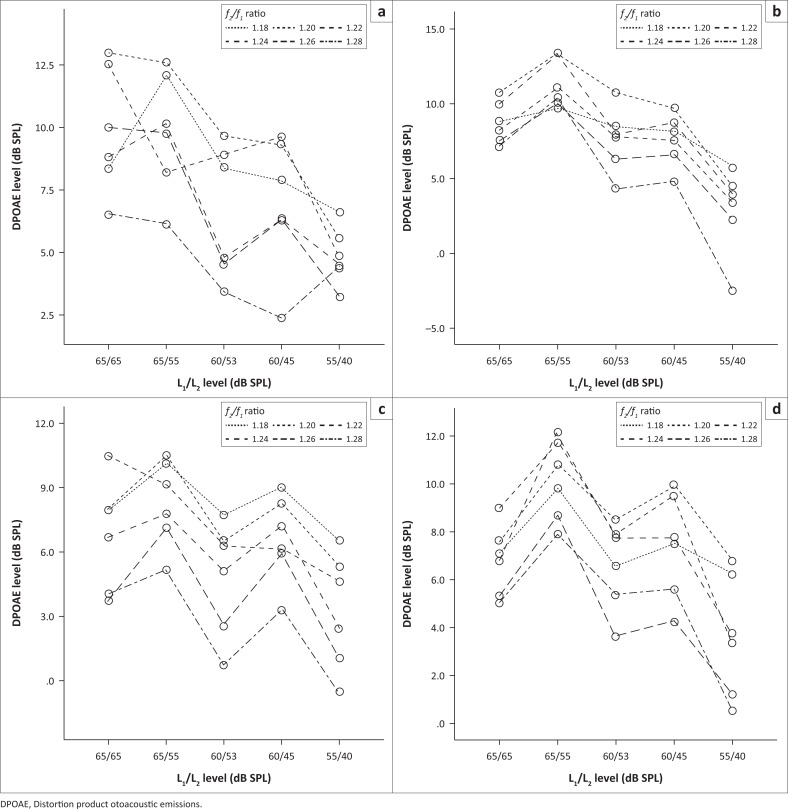

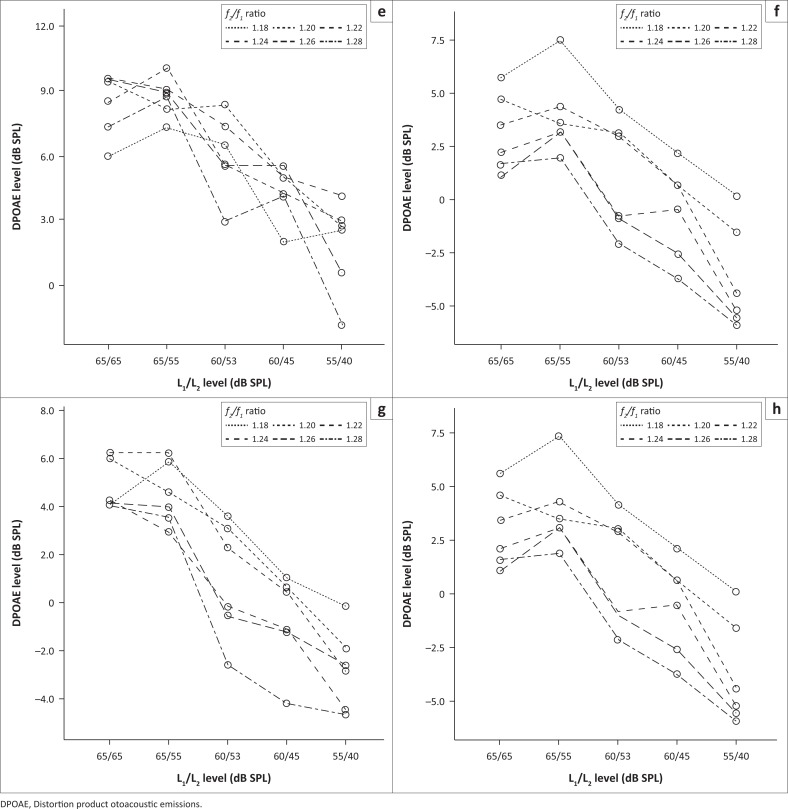

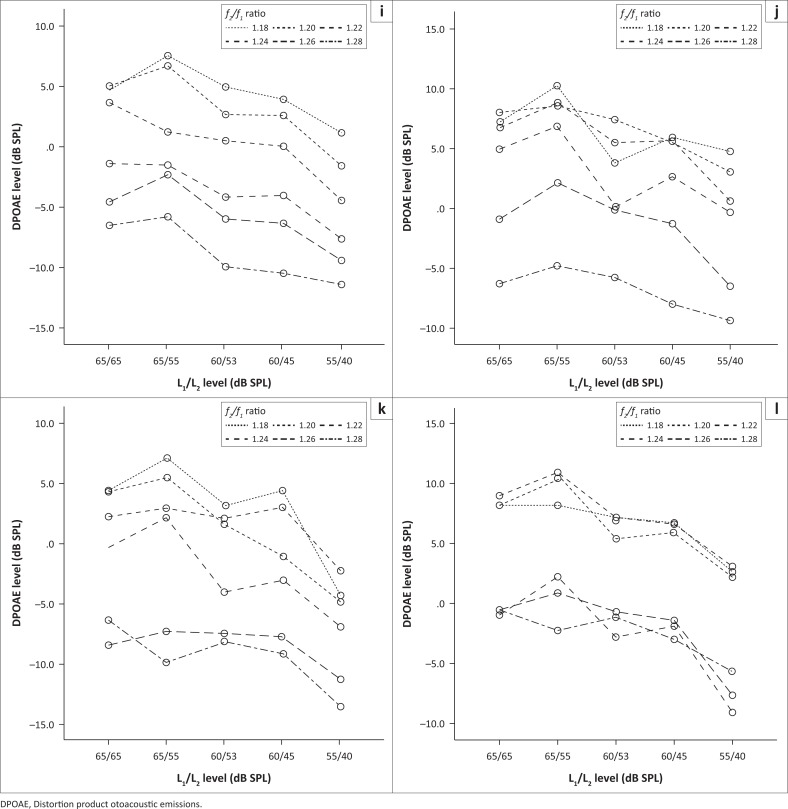

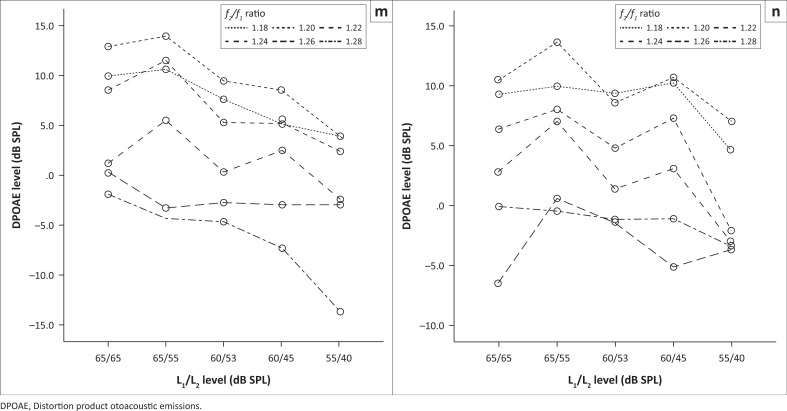
Mean distortion product otoacoustic emission absolute levels per intensity and frequency ratio combinations for each frequency (*f*_2_): (a) right ear at 2003 Hz, (b) left ear at 2003 Hz, (c) right ear at 2519 Hz, (d) left ear at 2519 Hz, (e) right ear at 3175 Hz, (f) left ear at 3175 Hz, (g) right ear at 3996 Hz, (h) left ear at 3996 Hz, (i) right ear 5000 Hz, (j) left ear at 5000 Hz, (k) right ear at 6996 Hz, (l) left ear at 6996 Hz, (m) right ear at 8003Hz and (n) left ear at 8003Hz. Hz, hertz.

Figure 1 shows the DPOAE mean absolute levels for all combinations of *f*_*2*_*/f*_*1*_ and L_1_/L_2_ at each *f*_2_ frequency for the participants at the first assessment occasion. This figure also presents the numbers of ears showing DPOAEs at each of these stimulus combinations. These results showed this study’s participants were more likely to show DPOAEs of higher intensity at lower *f*_2_ frequencies.

Table 1 shows the results of the linear mixed model analyses for main effects of level (L_1_/L_2_ in dB SPL) and frequency (*f*_*2*_*/f*_*1*_) settings. For all *f*_*2*_ values and in both ears, these analyses showed that the 65/55 and 65/65 level settings consistently resulted in higher DPOAE levels across all *f*_*2*_*/f*_*1*_ settings, and the 1.18, 1.20 and 1.22 *f*_*2*_*/f*_*1*_ settings regularly resulted in higher DPOAE levels across all L_1_/L_2_ settings.

**TABLE 1:** Results of the mixed model analyses for main effects (*p* < 0.05) of level (L_1_/L_2_ in dB SPL) and frequency (*f*_2_*/f*_1_) settings.

*f*_2_ (Hz)	Ear	Best L_1_/L_2_ (for all *f*_2_*/f*_1_ combined)	Best *f*_2_*/f*_1_ (for all L_1_/L_2_ combined)
2003	L	65/55	1.18, 1.20, 1.22
	R	65/55	1.20
2519	L	65/55	1.18, 1.20, 1.22
	R	65/55	1.18, 1.20, 1.22
3175	L	65/65, 65/55	1.18, 1.20, 1.22
	R	65/65, 65/55	1.20, 1.22
3996	L	65/65, 65/55	1.18, 1.20
	R	65/65, 65/55	1.18, 1.20, 1.22
5039	L	65/65, 65/55	1.18
	R	65/65, 65/55	1.18, 1.20
6351	L	65/65, 65/55	1.18, 1.20
	R	65/65, 65/55	1.18, 1.20, 1.22
8003	L	65/55	1.18
	R	65/65, /65/55	1.18, 1.20

L, left; R, right; SPL, sound pressure level.

Table 2 shows the results of the mixed model analyses of all level and frequency settings combined. For all *f*_*2*_ values and in both ears, these analyses showed that the level (dB SPL) and frequency ratio settings of 65/65 and 1.20, 65/55 and 1.22, 65/55 and 1.20, and 65/55 and 1.18 regularly resulted in higher DPOAE levels compared to other level and frequency ratio combinations.

**TABLE 2:** Results of the mixed model analyses of all level and frequency settings combined.

*f*_2_ (Hz)	Ear	Best combinations of L_1_/L_2_ and *f*_2_*/f*_1_ (only combinations showing a best result on at least one occasion are shown)													
		65/65					65/55					60/53		60/45	
		1.26	1.24	1.22	1.20	1.18	1.26	1.24	1.22	1.20	1.18	1.20	1.18	1.20	1.18
2003	L	-	-	-	X	-	-	X	X	X	X	-	-	-	-
	R	-	-	-	X	-	-	-	X	X	-	-	-	-	-
2519	L	-	-	-	-	-	X	X	X	X	X	-	-	-	-
	R	-	-	X	X	-	-	X	X	X	X	-	X	X	X
3175	L	-	X	X	X	-	-	X	X	X	-	-	-	-	-
	R	X	-	X	X	-	-	X	X	-	-	-	-	-	-
3996	L	-	-	X	X	-	-	-	X	X	X	X	X	-	-
	R	-	-	X	X	-	-	-	X	-	X	-	-	-	-
5039	L	-	-	-	X	X	-	-	-	X	X	-	-	-	-
	R	-	-	-	X	X	-	-	-	X	-	-	X	-	-
6351	L	-	-	X	X	X	-	-	X	-	X	-	-	-	-
	R	-	-	-	-	X	-	-	-	X	X	-	X	-	-
8003	L	-	-	-	-	X	-	-	-	X	X	-	X	-	-
	R	-	-	-	X	X	-	-	X	X	X	-	-	-	-
Counts	-	1	1	6	11	6	1	5	10	11	10	1	5	1	1

Note: Within each *f*_2_ and ear combination, the X’s indicate L_1_/L_2_ and *f*_2_*/f*_1_ stimulus combinations that produced distortion product otoacoustic emissions levels that were significantly (*p* < 0.05) higher than other L_1_/L_2_ and *f*_2_*/f*_1_ stimulus combinations in that row.

L, left; R, right.

The results of the ICC analysis of DPOAE results obtained for each *f*_*2*_ value, for right and left ears, and for every L_1_/L_2_ (dB SPL) and *f*_*2*_*/f*_*1*_ stimulus combination are not shown in this article (because of the very high number of these analyses conducted). Instead, Table 3 shows for each *f*_*2*_ value, for right and left ears, the lowest and highest ICC absolute agreement (single) coefficients with their 95% confidence intervals from all L_1_/L_2_ and *f*_*2*_*/f*_*1*_ stimulus combinations returning significant (*p* < 0.05) ICC values. Table 3 also shows for each *f*_*2*_ value, for right and left ears, the L_1_/L_2_ (dB SPL) and *f*_*2*_*/f*_*1*_ stimulus combinations that returned insignificant ICC values. No obvious patterns emerged regarding L_1_/L_2_ (dB SPL) and *f*_*2*_*/f*_*1*_ stimulus combinations that were more or less likely to return better or worse ICC results for each *f*_*2*_. It was noted, however, that more L_1_/L_2_ (dB SPL) and *f*_*2*_*/f*_*1*_ stimulus combinations returned insignificant ICC values for *f*_*2*_ = 8003 Hz, meaning that results at this frequency were more likely to be unreliable, regardless of the L_1_/L_2_ (dB SPL) and *f*_*2*_*/f*_*1*_ stimulus combinations used.

**TABLE 3:** Results of the intraclass correlation coefficient absolute agreement (single) analyses of distortion product otoacoustic emissions results obtained at each *f*_2_ value for each stimulus level (L_1_/L_2_ in dB SPL) and frequency (*f*_2_*/f*_1_) setting.

*f*_2_ (Hz)	Ear	ICC	For L_1_/L_2_ and *f*_2_*/f*_1_ stimulus combinations returning significant (*p* < 0.05) ICC values:			L_1_/L_2_ (dB SPL) and *f*_2_*/f*_1_ stimulus combinations returning insignificant (*p* > 0.05) ICC (single) values
			Lowest and highest ICC (single) coefficients	95% confidence intervals	L_1_/L_2_ (dB SPL) and *f*_2_*/f*_1_ stimulus combination	
2003	L	Highest	0.430	−0.02–0.73	65/65 and 1.22	65/65 and 1.20, 65/65 and 1.28, 65/55 and 1.22, 60/53 and 1.20, 55/40 and 1.20
		Lowest	0.790	0.53–0.92	60/45 and 1.28	
	R	Highest	0.410	−0.05–0.73	65/65 and 1.28	65/55 and 1.22, 55/40 and 1.22, 55/40 and 1.20, 55/40 and 1.28
		Lowest	0.910	0.78–0.96	65/55 and 1.20	
2519	L	Highest	0.390	−0.03–0.71	65/55 and 1.18	65/65 and 1.24
		Lowest	0.932	0.81–0.97	55/40 and 1.26	
	R	Highest	0.420	−0.01–0.72	65/55 and 1.18	55/40 and 1.28
		Lowest	0.880	0.73–0.95	60/53 and 1.18	
3175	L	Highest	0.260	−0.23–0.64	65/55 and 1.28	65/65 and 1.20, 65/65 and 1.22, 65/55 and 1.24, 60/53 and 1.18, 60/53 and 1.22, 60/45 and 1.24
		Lowest	0.630	0.26–0.85	55/40 and 1.22	
	R	Highest	0.430	−0.01–0.73	60/53 and 1.22	None
		Lowest	0.920	0.78–0.97	60/55 and 1.20	
3996	L	Highest	0.410	−0.05–0.73	65/55 and 1.18	65/55 and 1.26, 60/53 and 1.24, 55/40 and 1.28
		Lowest	0.900	0.73–0.96	60/45 and 1.26	
	R	Highest	0.380	−0.06–0.70	65/65 and 1.26	65/55 and 1.28, 60/53 and 1.24, 60/45 and 1.28, 55/40 and 1.28
		Lowest	0.920	0.80–0.97	60/53 and 1.18	
5039	L	Highest	0.540	0.08–0.81	65/65 and 1.20	60/53 and 1.18
		Lowest	0.940	0.77–0.99	55/40 and 1.26	
	R	Highest	0.340	−0.17–0.71	60/53 and 1.22	60/45 and 1.28, 55/40 and 1.28
		Lowest	0.930	0.81–0.98	60/45 and 1.22	
6351	L	Highest	0.490	−0.10–0.83	65/55 and 1.26	60/53 and 1.26, 60/45 and 1.26, 60/45 and 1.28
		Lowest	0.930	0.67–0.99	55/40 and 1.18	
	R	Highest	0.500	−0.11–0.84	60/53 and 1.22	65/55 and 1.24, 60/53 and 1.26, 60/45 and 1.24, 60/45 and 1.26
		Lowest	0.940	0.84–0.98	65/65 and 1.18	
8003	L	Highest	0.470	0.01–0.77	60/53 and 1.18	65/65 and 1.18, 65/65 and 1.26, 65/55 and 1.28, 60/53 and 1.20, 60/53 and 1.24, 60/53 and 1.26, 60/53 and 1.28, 60/45 and 1.20, 60/45 and 1.26, 60/45 and 1.28, 55/40 and 1.18, 55/40 and 1.24, 55/40 and 1.26, 55/40 and 1.28
		Lowest	0.870	0.56–0.97	55/40 and 1.22	
	R	Highest	0.430	−0.08–0.77	65/55 and 1.22	65/65 and 1.24, 65/65 and 1.26, 65/55 and 1.28, 60/53 and 1.24, 60/53 and 1.28, 60/45 and 1.24, 60/45 and 1.28, 55/40 and 1.22, 55/40 and 1.26, 55/40 and 1.28
		Lowest	0.990	0.79–1.00	60/53 and 1.26	

ICC, intraclass correlation coefficient; L, Left; R, Right; SPL, sound pressure level.

## Discussion

Overall, the L_1_/L_2_ combinations and *f*_*2*_*/f*_*1*_ ratios used in this study elicited DPOAEs of varying amplitude and reliability. An L_1_/L_2_ combination of 65/55 dB SPL appeared to elicit the largest DPOAEs at most *f*_*2*_ values, followed by an L_1_/L_2_ combination of 65/65. This finding supports similar findings regarding the L_1_/L_2_ combinations more likely to elicit larger DPOAEs from human adults (Beattie & Jones, 1998; Vento, Durrant, Sabo, & Boston, 2004). Direct comparisons between this study’s findings and similar studies in the literature were difficult, however, with many studies in the literature having used higher L_1_/L_2_ levels than this study (Beattie, Kenworthy, & Neal-Johnson, 2004; Hauser & Probst, 1991; Meinke et al., 2013; Whitehead, McCoy, Lonsbury-Martin, & Martin, 1995). These higher L_1_/L_2_ levels were avoided in this study because of their higher likelihood of eliciting false-negative results and artefacts (Dhar & Hall, 2012).

The *f*_*2*_*/f*_*1*_ ratios of 1.18, 1.20 and 1.22 appeared to elicit the largest DPOAEs at most *f*_*2*_ values. This finding supports similar findings regarding the *f*_*2*_*/f*_*1*_ ratios that are more likely to elicit larger DPOAEs from human adults (Abdala, 1996; Dreisbach & Siegel, 2001; Gaskill & Brown, 1990) as well as supporting previous reports that the best *f*_*2*_*/f*_*1*_ ratio appears to decrease as *f*_*2*_ increases and vice versa (Abdala, 1996; Dreisbach & Siegel, 2001).

Stimulus parameters using an L_1_/L_2_ of 65/65 with an *f*_*2*_*/f*_*1*_ ratio of 1.20 or an L_1_/L_2_ of 65/55 with *f*_*2*_*/f*_*1*_ ratios of 1.18, 1.20 or 1.22 appeared to elicit the largest DPOAEs at most *f*_*2*_ values. This result supports similar findings regarding the L_1_/L_2_ and *f*_*2*_*/f*_*1*_ ratio parameter settings that are more likely to elicit larger DPOAEs from human adults (Beattie & Jones, 1998; Vento et al., 2004).

This study’s results do not explain why the largest DPOAEs were elicited using stimulus parameters using L_1_/L_2_ combinations of 65/65 dB SPL or 65/55 dB SPL and *f*_*2*_*/f*_*1*_ ratios of 1.18, 1.20 or 1.22. Regarding L_1_/L_2_ combinations, the larger DPOAEs elicited by stimuli with primaries of 65 (i.e. the 65/65 dB SPL and 65/55 dB SPL level stimuli) could be related to the function of the cochlear amplifier (Harris et al., 1989) as stimuli, with lower level primaries (L_1_/L_2_ levels of 60/53 dB SPL, 60/45 dB SPL and 55/40 dB SPL) yielding lower level DPOAEs. Such a possibility would be generally consistent with Brown and Gaskill (1990) who reported DPOAE amplitude to depend more on the level of L_1_ than L_2_. Regarding *f*_*2*_*/f*_*1*_ ratios, the larger DPOAEs elicited by *f*_*2*_*/f*_*1*_ ratios of 1.18, 1.20 or 1.22 could reflect the cochlea’s frequency selectivity and bandpass filter function or properties (Allen & Fahey, 1993). Such a possibility would be generally consistent with Gaskill and Brown (1990) and Harris et al. (1989) who found that DPOAE levels peaked at *f*_*2*_*/f*_*1*_ ratios of 1.22 and 1.25, respectively, with a decline with higher or lower *f*_*2*_*/f*_*1*_ ratios. Stover, Neely, and Gorga (1999) suggested that these declines at higher *f*_*2*_*/f*_*1*_ ratios could result from greater separation of the primaries that lessen the interaction of their travelling waves on the basilar membrane, whereas the declines at lower *f*_*2*_*/f*_*1*_ ratios could result from less separation of the primaries and greater cancellation of their travelling waves on the basilar membrane.

Although some L_1_/L_2_ combinations and *f*_*2*_*/f*_*1*_ ratios clearly elicited larger DPOAEs, no L_1_/L_2_ combinations and *f*_*2*_*/f*_*1*_ ratios clearly elicited more reliable DPOAEs. This was consistent with previous reports that commonly used sets of stimulus parameters to elicit DPOAEs of similarly varying reliability (Stuart, Passmore, Culbertson, & Jones, 2009; Wagner, Heppelmann, Vonthein, & Zenner, 2008) but inconsistent with reports finding higher L_1_/L_2_ combinations to elicit more reliable DPOAEs (Franklin, McCoy, Martin, & Lonsbury-Martin, 1992; Keppler et al., 2010; Roede, Harris, Probst, & Xu, 1993). It must be noted that DPOAEs for *f*_*2*_ = 8003 Hz in this study were most likely to be unreliable. This finding could indicate that any DPOAE recorded at such high *f*_*2*_ frequencies is likely to be unreliable; however, such a conclusion should be interpreted with caution as varying the location of the probe microphone has been shown to affect the calibration of the sound source at these frequencies (Siegel, 2002).

## Conclusion

The study concluded that further, targeted investigation of the 65/65 dB SPL, 65/55 dB SPL and 60/53 dB SPL intensity levels and the 1.18, 1.20, 1.22 *f*_*2*_*/f*_*1*_ ratios is warranted to determine the best stimulus parameters for eliciting the largest and most reliable DPOAEs in adult humans. In addition, these stimulus parameters should be investigated in individuals with hearing loss of cochlear origin to select the parameters most sensitive to cochlear damage.
